# The Integrin‐Mediated ILK‐Parvin‐αPix Signaling Axis Controls Differentiation in Mammary Epithelial Cells

**DOI:** 10.1002/jcp.25390

**Published:** 2016-06-10

**Authors:** Nicholas Rooney, Pengbo Wang, Keith Brennan, Andrew P. Gilmore, Charles H. Streuli

**Affiliations:** ^1^ The Wellcome Trust Centre for Cell‐Matrix Research and Manchester Breast CentreFaculty of Life SciencesUniversity of ManchesterManchesterUnited Kingdom

## Abstract

Epithelial cell adhesion to the surrounding extracellular matrix is necessary for their proper behavior and function. During pregnancy and lactation, mammary epithelial cells (MECs) receive signals from their interaction with laminin via β1‐integrin (β1‐itg) to establish apico‐basal polarity and to differentiate in response to prolactin. Downstream of β1‐itg, the scaffold protein Integrin Linked Kinase (ILK) has been identified as the key signal transducer that is required for both lactational differentiation and the establishment of apico‐basal polarity. ILK is an adaptor protein that forms the IPP complex with PINCH and Parvins, which are central to its adaptor functions. However, it is not known how ILK and its interacting partners control tissue‐specific gene expression. Expression of ILK mutants, which weaken the interaction between ILK and Parvin, revealed that Parvins have a role in mammary epithelial differentiation. This conclusion was supported by shRNA‐mediated knockdown of the Parvins. In addition, shRNA knockdown of the Parvin‐binding guanine nucleotide exchange factor αPix prevented prolactin‐induced differentiation. αPix depletion did not disrupt focal adhesions, MEC proliferation, or polarity. This suggests that αPix represents a differentiation‐specific bifurcation point in β1‐itg‐ILK adhesive signaling. In summary, this study has identified a new role for Parvin and αPix downstream of the integrin‐ILK signaling axis for MEC differentiation. J. Cell. Physiol. 231: 2408–2417, 2016. © 2016 The Authors. *Journal of Cellular Physiology* Published by Wiley Periodicals, Inc.

Cells in multicellular organisms require signals from multiple sources, which cooperate to control cell fate decisions and differentiation into tissue‐specific cell types with unique functions. The mammary gland undergoes regulated and defined morphological and functional changes during adulthood (Watson and Khaled, 2008). For instance, during pregnancy the anterior pituitary gland produces a 22 kDa peptide hormone prolactin (Prl), which acts on the mammary gland to induce differentiation (Freeman et al., 2000). Prl causes the formation of lobuloalveolar units containing terminally differentiated MECs capable of milk production that exist in collections of rounded, hollow acini at tips of branched collecting ducts (Oakes et al., 2008; Bernichtein et al., 2010; Shehata et al., 2012).

While hormones temporally direct mammary gland development, there is also a fundamental requirement for integrin‐mediated ECM adhesion in MEC behavior (Muschler and Streuli, 2010; Glukhova and Streuli, 2013). It is established that β1‐itg mediated adhesion is required for the progression of MECs through the cell cycle and the establishment of apico‐basal polarity in these cells (Li et al., 2005; Naylor et al., 2005). During pregnancy, Prl initiates an integrin‐dependent Jak/Stat signaling cascade that results in the transcription of milk protein genes including β‐casein, a marker of terminal MEC differentiation (Gouilleux et al., 1994; Lebrun et al., 1994; Pfitzner et al., 1998).

ILK is a 50 kDa multi‐domain scaffold protein that mediates protein‐protein interactions between ILK‐binding partners (Hannigan et al., 1996; Rooney and Streuli, 2011; Widmaier et al., 2012). Central to ILK's scaffold function is its existence in an IPP complex bound by PINCH and Parvin. The IPP complex coordinates downstream effectors such as GEFs, GAPs and kinases around integrin tails. ILK is involved in different cellular processes, the importance of which is highlighted in vivo by the embryonic lethality of ILK‐null mice (Sakai et al., 2003). Moreover in the mammary gland, analysis of ILK‐null MECs showed that polarized acini failed to form, lactation was reduced, and in vivo pups were undersized and malnourished (Akhtar et al., 2009; Akhtar and Streuli, 2013). However, it is not known what ILK associates with in order to transmit the adhesive cues from β1‐itg that are necessary for epithelial differentiation.

In this study, we hypothesized that specific ILK‐binding partners link integrins to the prolactin‐triggered differentiation programme in mammary epithelia (Rooney and Streuli, 2011). We found that ILK mutants unable to bind Parvin, and shRNAs to the Parvins, suppressed MEC differentiation. In addition, shRNA knockdown of the Parvin‐interacting protein, αPix, revealed that this protein was specifically required for MEC differentiation, while not affecting other key MEC behaviors. Our data suggest that the ILK‐Parvin‐Pix signaling axis is important for tissue‐specific gene expression in the mammary gland.

## Results

### Parvins have a role in mammary epithelial cell differentiation

**Figure 1:**
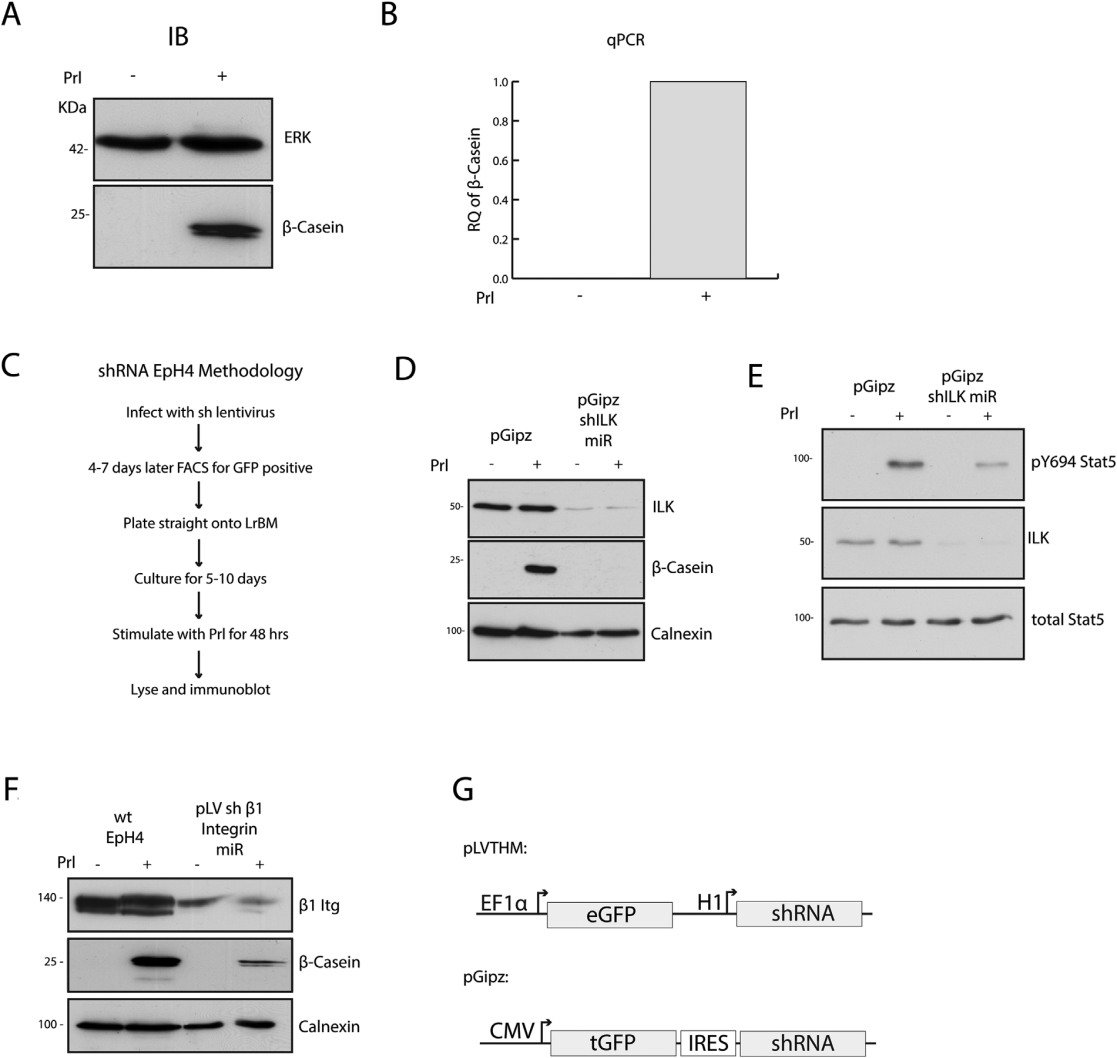
EpH4 MECs differentiate when treated with Prolactin and require ILK and β1Itg. (A‐B) EpH4s cultured on LrBM produce β‐casein only when stimulated with Prl, as detectable by immunoblot against β‐casein (A) and qPCR analysis of β‐casein mRNA expression (B). RQ = relative quantification. (C) Standard methodology for all lentiviral shRNA differentiation studies in EpH4s. (D) ILK is knocked down in EpH4s infected with pGipz shILK miR in comparison to EpH4s infected with pGipz control vector. shILK EpH4s fail to respond to Prl and don't produce β‐casein. (E) shILK EpH4 MECs were cultured as in (D), but were stimulated with Prl for 15 min. Assessed by immunoblot for Stat5 phospho‐Y694, shILK EpH4's have reduced phosphorylation of Y694 in response to Prl compared to pGipz EpH4 cells. (F) EpH4s infected with pLVTHM shβ1Itg miR were cultured as outlined in (C). shβ1Itg EpH4s have reduced levels of β1Itg, and β‐casein after Prl stimulation. (G) Linear vector maps of pGipz and pLVTHM shRNA expression vectors.

In order to study the role of ILK‐regulated proteins in the control of Prl‐driven differentiation, we used the mouse MEC cell line EpH4, which was originally isolated from mid‐pregnant mice (Fialka et al., 1996). To induce differentiation, MECs were cultured on 3D LrBM and treated with the lactogenic hormone Prl (Fig. 1A and B). Lentiviral delivery of shRNA miRs targeting ILK or β1‐Itg caused MECs to produce lower levels β‐casein and reduced levels of transiently phosphorylated Stat5‐Y694 (Fig. 1C–G). This confirmed the role of β1‐itg:ILK signaling in EpH4s, and established the utility of the EpH4 cell line as a MEC differentiation model (Naylor et al., 2005; Akhtar et al., 2009).

**Figure 2:**
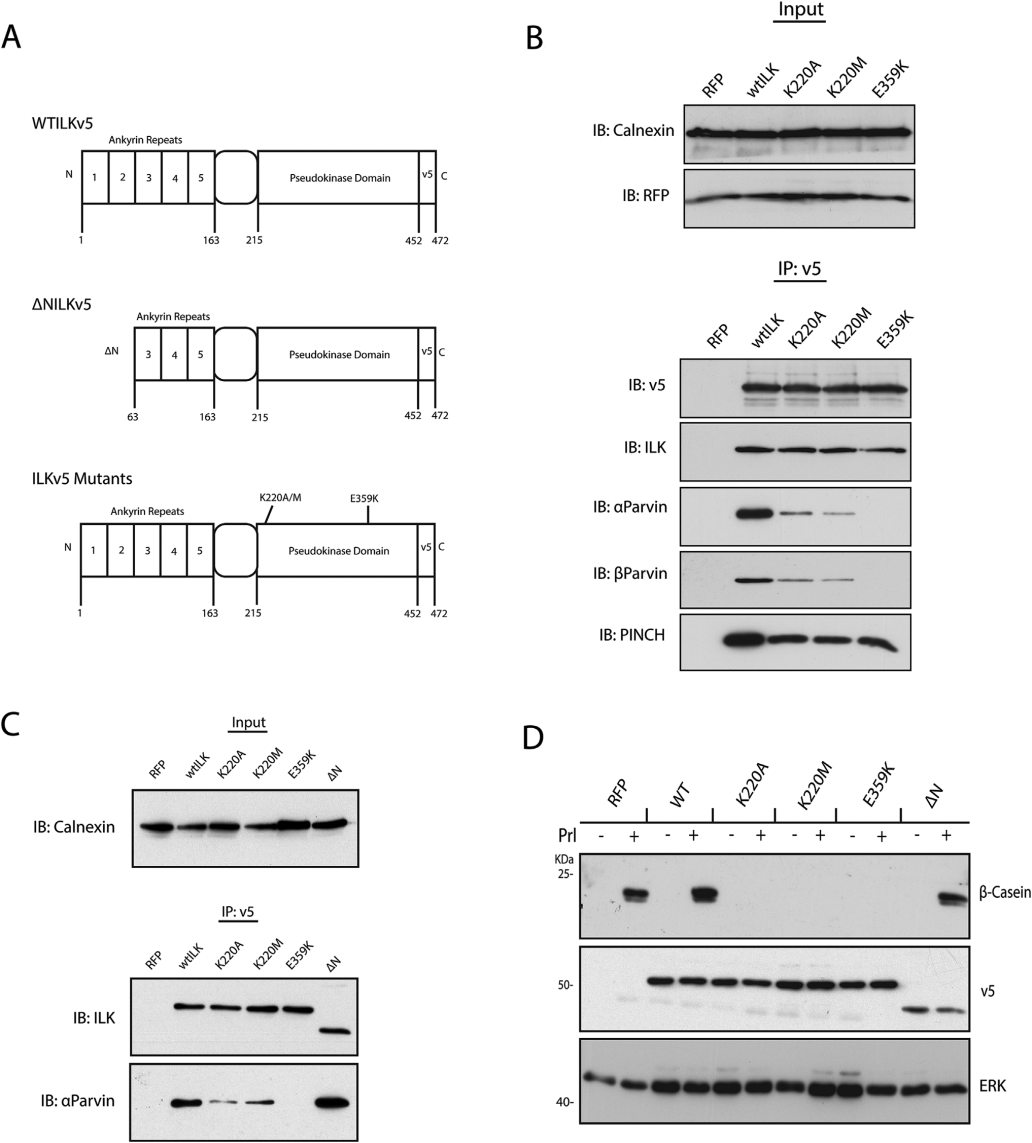
EpH4 cells that express ILK mutants that do not bind to Parvin fail to differentiate. (A) Schematic of the ΔN truncation and point mutations in the ILKv5 mutant constructs stably expressed in EpH4 cell lines. (B) Immunoprecipitation of the v5 tag and subsequent immunoblotting for αParvin, βParvin and PINCH. The K220A/K220M mutants have reduced Parvin binding and the E359 K mutant did not bind at all, while binding to PINCH remained in each case. Immunoblotting the IP complex for ILK and v5 confirmed similar IP levels and IB of the total lysate (input) confirmed equal starting material. (C) EpH4s expressing ILK mutants were immuno‐precipitated for the v5 tag with subsequent immunoblotting for αParvin. K220A/K220M mutants have reduced Parvin binding and the E359 K mutant did not bind, while ΔN‐ILKv5 still binds to Parvin at levels similar to wt. Immunoblotting the IP complex for ILK confirmed similar IP levels and IB of the total lysate (input) for Calnexin confirmed equal starting material. (D) ILKv5 mutant EpH4 cell lines were cultured on LrBM prior to differentiation by the addition of Prl for 48 h. Representative immunoblots against β‐casein showed that the wtILKv5 and ΔN‐ILKv5 expressing EpH4's produced β‐casein equivalent to mRFP control cells. K220A, K220M, and E359 K mutant EpH4s failed to produce β‐casein. Immunoblots against v5 confirms expression of the ILKv5 mutants and Calnexin is a loading control. The cell lines were independently generated twice.

ILK provides a scaffold for protein‐protein interactions at integrin tails, around which the IPP complex forms (Rooney and Streuli, 2011). However, deletion of ILK precludes investigation into the role of its binding partners as both parvin and PINCH are dependant on ILK for their stability (Fukuda et al., 2003). To determine the role of ILK's interacting partners in MEC differentiation, altered versions of ILK, encoding point mutations or a truncation, were lentivirally‐introduced into MECs, and cell lines were established (Fig. 2A).

K220A and K220M are known to prevent Parvin binding (Yamaji et al., 2001; Filipenko et al., 2005; Lange et al., 2009; Fukuda et al., 2011), while an N‐terminal truncation specifically prevents binding to PINCH only (Li et al., 1999; Lynch et al., 1999; Tu et al., 1999; Chiswell et al., 2008; Yang et al., 2009) and E359 K has been described as a “dominant negative” mutation (Persad et al., 2001; Nikolopoulos and Turner, 2002; Filipenko et al., 2005; Lange et al., 2009). To confirm the effect of these mutations on ILK‐binding partners, the C‐terminal v5 tag of ILKv5 was immunoprecipitated from lysates of control (mRFP only) and wt and mutant ILKv5 cell lines. The K220 and E359 K mutants showed either reduced or completely ablated binding to α‐ and β‐Parvins, while they were still able to bind to PINCH (Fig. 2B). The ΔN‐ILKv5 truncation was still able to bind to Parvin (Fig. 2C).

ILK mutant MECs were induced to differentiate on 3D LrBM culture by the addition of Prl, and β‐casein production was assayed by immunoblot. In comparison to control cells expressing mRFP only, wtILKv5, or ΔN‐ILKv5, the MECs expressing the K220A, K220M, and E359 K ILK mutants did not produce β‐casein (Fig. 2D). These data indicate that ILK mutants with disrupted ILK‐Parvin interactions, but with normal ILK‐PINCH binding, prevented the ability of MECs to differentiate and express β‐casein in response to Prl.

To confirm a role for Parvin in Prl driven differentiation, we developed shRNA hairpins that knockdown specific ILK partners, including α‐ and β‐Parvin. MECs were transduced with lentiviruses expressing shRNAs together with GFP. FACS‐sorted, homogenous GFP‐positive MECs were plated on 3D LrBM for subsequent Prl treatment and differentiation assays (as outlined in Fig. 1C).

**Figure 3:**
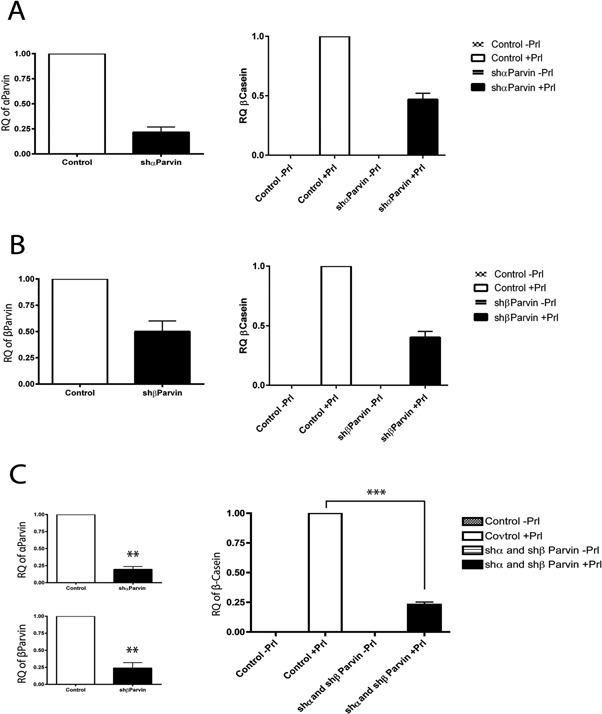
Knockdown of Parvins causes a differentiation defect. EpH4's infected with either control vector, shParvin or shβParvin or both shαParvin and shβParvin together were FAC sorted, and the cells were differentiated by the standard method prior to RNA extraction 48 h after Prl treatment. (A) Differentiated shαParvin cells had a 78% reduction in αParvin expression and a 53% reduction in β‐casein expression compared to control cells. (B) Differentiated sh βParvin cells had a 50% reduction in βParvin and a 60% reduction in β‐casein expression compared to control cells. (C) In EpH4s infected with both shαParvin and shβParvin lentiviruses, αParvin was reduced by 81% and βParvin was reduced by 76%, respectively. Following differentiation, β‐casein expression was reduced by 77% compared to control cells. Graphs present mean +/− SEM, *P* = < 0.003 = ** for α and βParvin knockdown, *t*‐test. *P* = < 0.005 = *** one way ANOVA for β‐casein expression. RQ = relative quantification.

MECs with reduced expression of Parvin showed reduced β‐casein production in response to Prl (Fig. 3A and B). Reduction in either α‐ or β‐Parvin caused a partial reduction in β‐casein production. However, simultaneous depletion of both α‐ and β‐Parvin had a greater effect on differentiation and caused a more substantial decrease in β‐casein production (Fig. 3C).

These results reveal that the ILK binding partners, α‐ and β‐Parvins, have a role in controlling the differentiation of MECs.

### αPix is required for mammary epithelial cell differentiation

**Figure 4:**
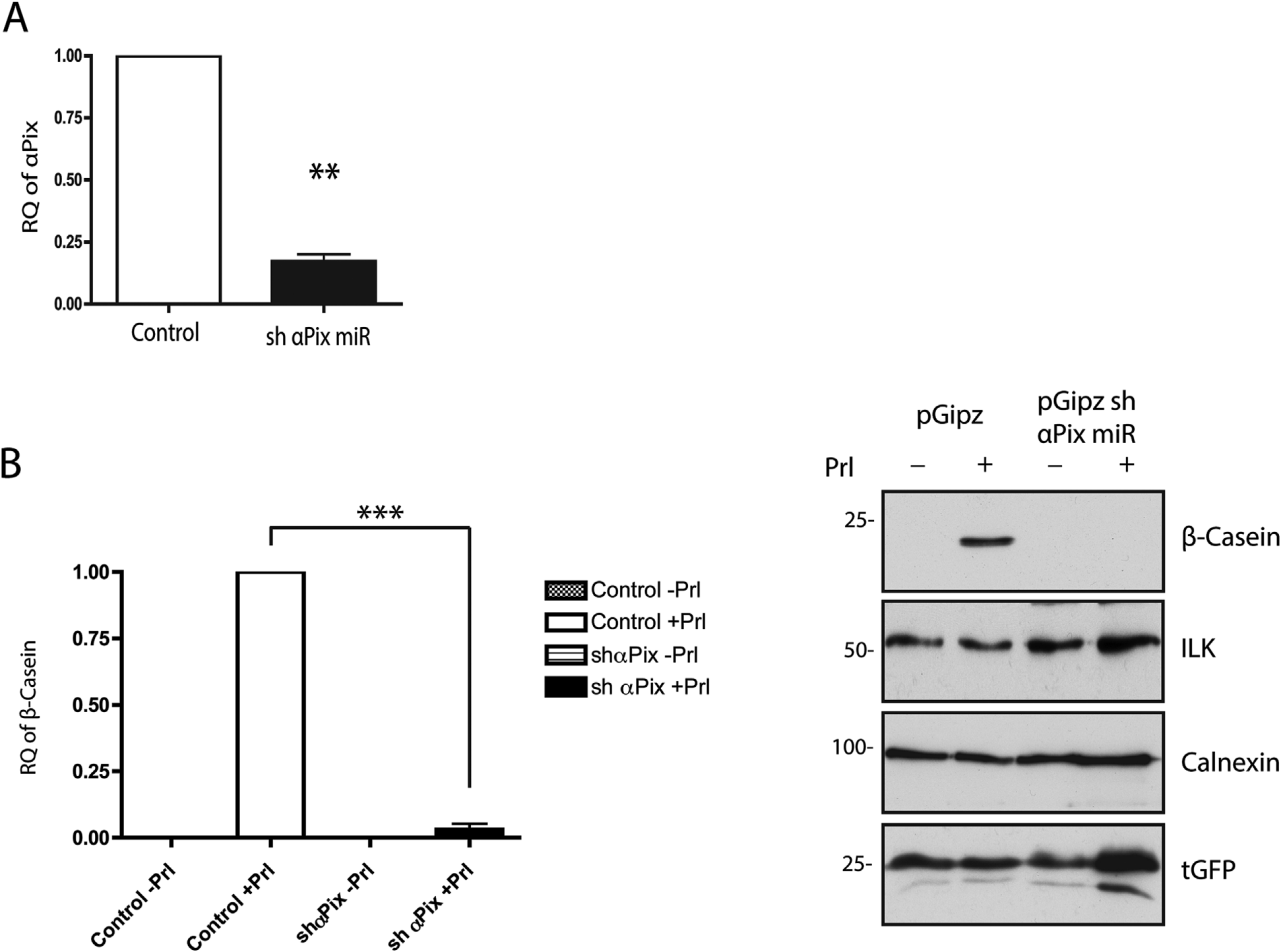
Knockdown of αPix in EpH4 cells causes a differentiation defect. EpH4 cells infected with pGipz or pGipz shαPix miR were FACs isolated and differentiated in LrBM culture. (A) αPix expression was significantly reduced by 83% in shαPix cells as quantified by qPCR (n = 3, graphs represent mean +/− SEM, *P* = 0.0012 = ** *t*‐test). (B) From the same samples used in (A) control cells stimulated with Prl produced detectable levels of β‐casein mRNA, normalized to 1. shαPix cells treated with Prl showed a 96% reduction in expression levels of β‐casein (n = 3, graphs represent mean +/− SEM, *P* = 0.0001 = *** one way ANOVA). In parallel differentiation experiments, pGipz EpH4 control cells express β‐casein in response to Prl (detected by IB), while shαPix miR EpH4 cells do not produce any detectable β‐casein. All samples express tGFP and ILK protein levels are not affected by αPix depletion. RQ = relative quantification.

Since αPix associates with ILK and the IPP complex via interactions with α‐Parvin, we reasoned that αPix may also have a role in MEC differentiation (Mishima et al., 2004; Filipenko et al., 2005). To test this hypothesis, shRNA hairpins that knockdown αPix were introduced into MECs followed by FAC sorting and cell culture on 3D LrBM. We examined whether αPix depletion affected mammary epithelial differentiation. Analysis by qPCR and immunoblot revealed that the shαPix‐depleted cells failed to produce β‐casein in response to Prl stimulation (Fig. 4A and B).

**Figure 5:**
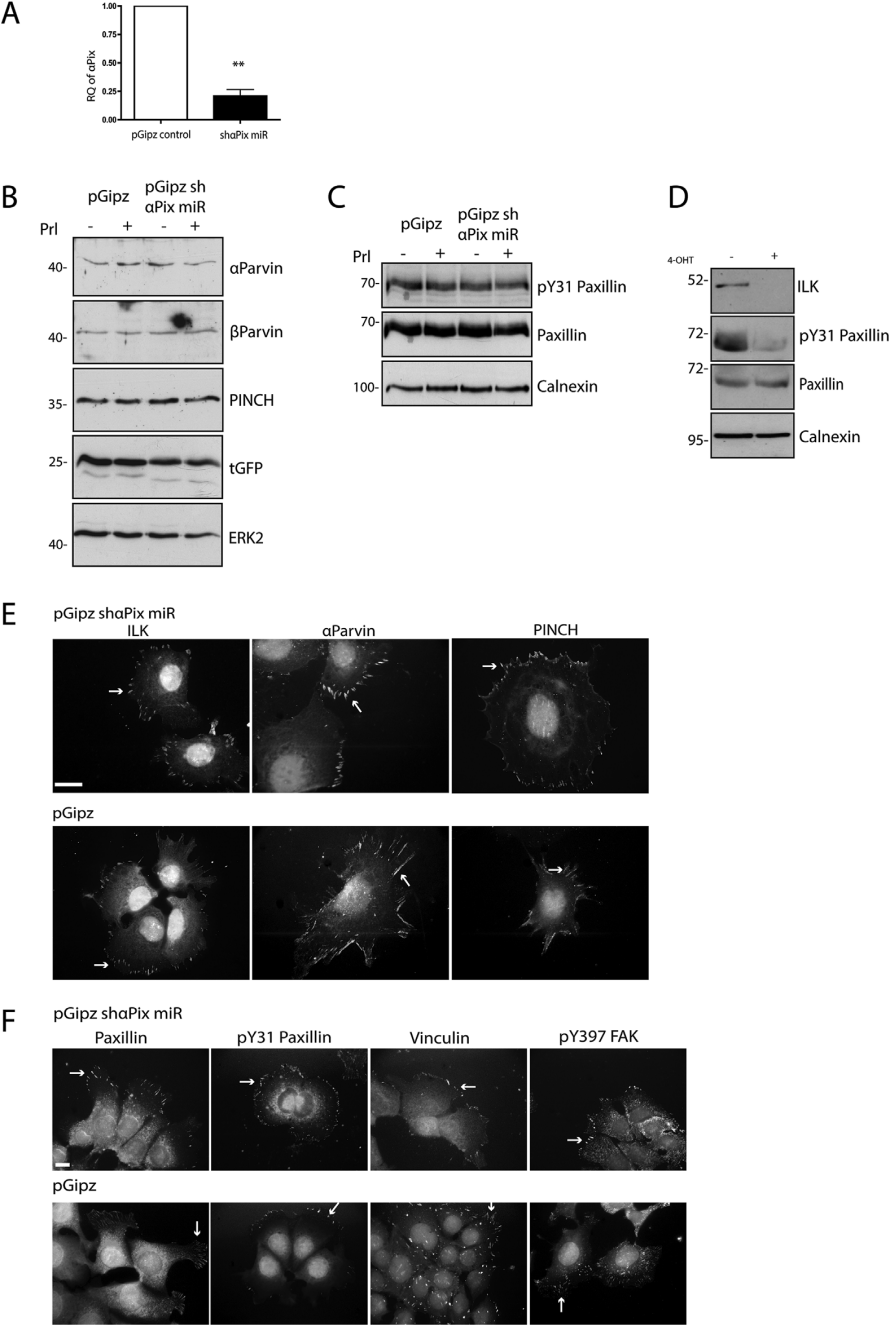
Knockdown of αPix does not disrupt IPP complex localisation and protein levels. EpH4s infected with either pGipz control or pGipz sh αPix miR were FAC sorted and plated directly onto coverslips. The cells were immuno‐stained for IPP and focal adhesion proteins. (A) qPCR from parallel 2D cultures showed an 80% reduction in αPix expression in shαPix miR cells compared to pGipz control cells, n = 3, graph represents mean +/− SEM, **P = 0.0052 *t*‐test. RQ = relative quantification. (B) Immunoblots showed protein levels of αParvin, βParvin and PINCH were not altered in shαPix EpH4s compared to control EpH4's. (C) Immunoblots show levels of both total Paxillin and pY31 Paxillin were not affected in shαPix EpH4s when compared to control EpH4's. (D) Immunoblots from ILK‐flox primary MECs show that pY31 Paxillin levels were reduced when ILK was depleted with 4‐OHT (4‐hydroxtamoxifen). (E) ILK, Parvin and PINCH localized to focal adhesions in pGipz shαPix miR cells as in control cells, indicated by arrows. (F) Paxillin, pY31Paxillin, Vinculin and pY397FAK localized to focal adhesions irrespective of shαPix miR expression, indicated by arrows, scale = 10 µm.

Next, we determined whether knockdown of αPix altered the levels of IPP proteins (Fig. 5A). There was no change in the levels of ILK, α/β Parvin or PINCH in comparison to controls (Fig. 4B, 5B). Additionally, the levels of pY31Paxillin were not altered in shαPix MECs compared to control cells (Fig. 5C), while pY31 was reduced when ILK was depleted from MECs (Fig. 5D). To expand on this result, we also evaluated whether αPix depletion altered the localisation of IPP proteins within adhesion complexes. Immunostaining FACS‐sorted shαPix MECs revealed ILK, Parvin and PINCH within focal adhesions, similar to control cells (Fig. 5E). Other adhesion proteins, Paxillin and Vinculin, also localized normally in αPix‐depleted cells, and there was similar localisation of the ILK‐dependent pY31Paxillin and adhesion‐dependent pY397FAK in the control and αPix‐depleted cells (Fig. 5F). Together these results indicate that αPix depletion does not affect the levels or localisation of IPP complex proteins, or cause a disruption to cellular adhesion complexes.

**Figure 6:**
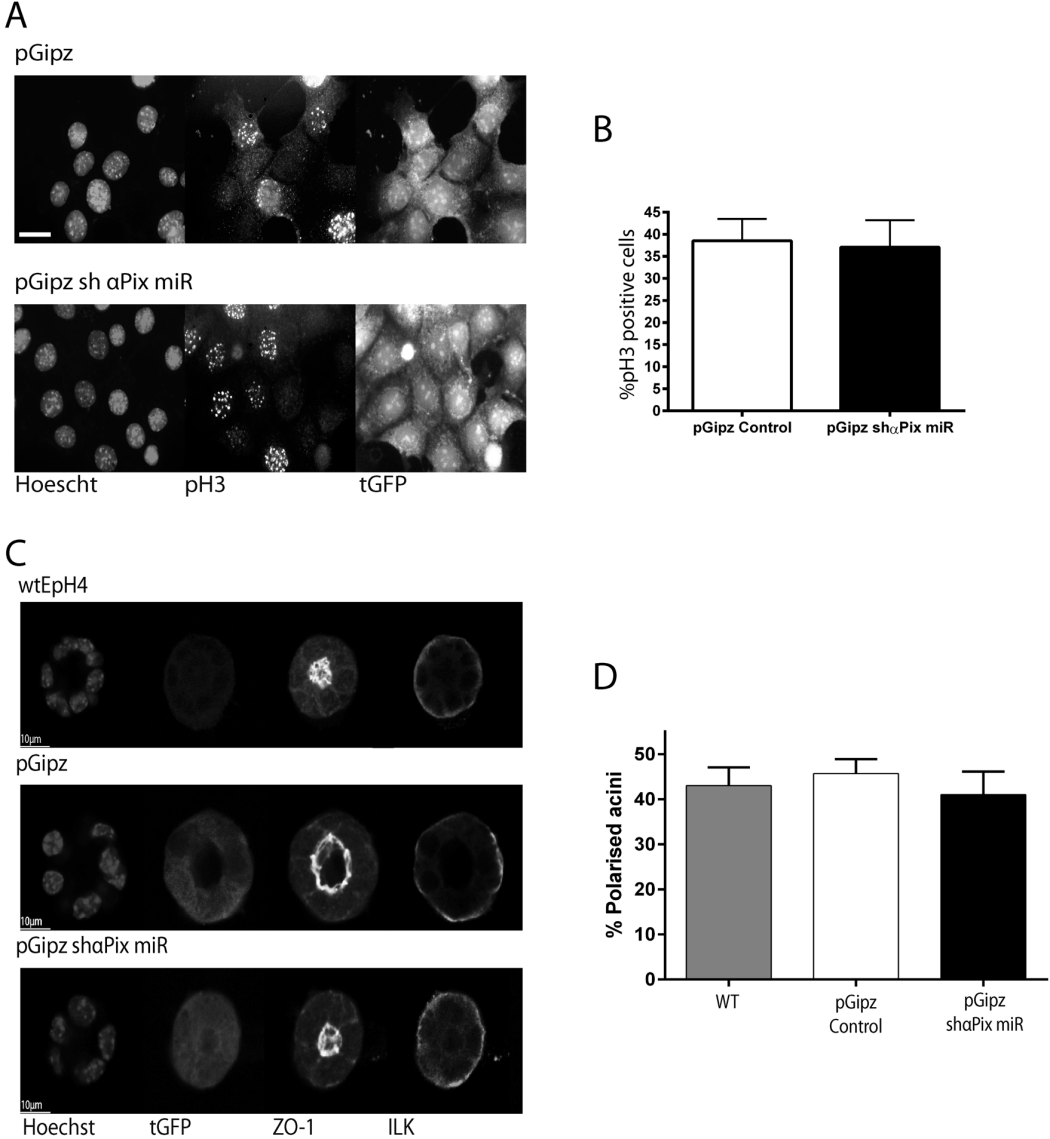
Knockdown of αPix does not affect MEC proliferation or polarity. (A) FAC sorted pGipz and pGipz shαPix miR EpH4s were plated onto 2D coverslips and immuno‐stained for phospho‐Histone 3 (pH3). Representative images show pH3 staining in the nucleus of cells undergoing mitosis. (B) Quantification of the % of pH3 positive cells showed a similar level of proliferation between pGipz (39%) and pGipz shαPix miR infected EpH4s (37%). Quantification is average of n = 3, −/+SD. The cells used are from the same 2D populations used in Figure 5, scale = 20 µm. (C) WT and FAC sorted pGipz control and pGipz shαPix miR EpH4s were plated onto LrBM coated coverslips. The cells were cultured for 7 days prior to being immuno‐stained for ILK, ZO‐1 and tGFP. Representative confocal pictures through 3D‐acini show that all cultures contained some polarized acini, with ILK around the basal edge and ZO‐1 at the apical domain. (D) Quantification of the % of polarized acini from separate experiments found similar levels of polarisation, ranging from 41–46% (average 25 acini counted per condition). αPix was depleted as in Figure 4A.

To explore the mechanism linking αPix with the expression of milk proteins, we examined whether αPix depletion altered other fundamental adhesion‐dependent behaviors that could influence the ability of MECs to differentiate. Adhesion signaling controls cell cycle in MECs (Jeanes et al., 2012), so we determined whether a reduction in αPix affects MEC proliferation by quantifying the percent of phospho‐Histone H3 (pH3) positive cells compared to control cells (Hans and Dimitrov, 2001). FACS‐sorted pGipz control and pGipz shαPix miR MECs cultured in the presence of serum showed a similar proportion of cells staining positive for pH3 in both control and αPix depleted cells (Fig. 6A and B). Thus the differentiation defect is unlikely to be caused by an effect of αPix on proliferation.

Adhesion signaling is required for the ability of MECs to form polarized acini (Akhtar and Streuli, 2013), so we assessed whether failure to differentiate is associated with a polarisation defect. FACS‐sorted shαPix cells (isolated at the same time as for the milk expression studies) were cultured on LrBM and analysed by confocal microscopy. Polarized acini were found in all conditions, as shown by staining for the apical marker ZO‐1 and the basal marker ILK (Fig. 6C). Quantification of the number of polarized acini revealed that polarisation was not effected in the shPix miR‐expressing cells (Fig. 6D). Thus the differentiation defect caused by αPix depletion is not caused by defective polarization.

These results show that αPix has a role in the control of MEC differentiation. It does not appear to be required for other key adhesion dependent MEC behaviors such as polarity or proliferation.

## Discussion

Signals from the ECM have long been known to influence tissue‐specific behavior in MECs, however the mechanisms are not fully understood. When MECs isolated from pregnant mice are stimulated with prolactin on 3D LrBM, they retain in vivo characteristics and differentiate to produce milk (Aggeler et al., 1991; Streuli et al., 1991). Genetic deletion of either β1‐integrins or ILK has revealed a key requirement for these proteins in transduction of the adhesive contribution to MEC differentiation (Naylor et al., 2005; Akhtar et al., 2009). In the present study, we demonstrate a role for the Parvins in the differentiation ability of MECs. Moreover MECs with reduced expression of αPix fail to differentiate, while maintaining otherwise normal MEC behavior. This represents both the discovery of a novel function for the αPix protein and the identification of Pix as a node downstream of the integrin‐ILK signaling axis for cellular differentiation.

### αPix is a new component of adhesion signaling in MEC differentiation

αPix (ARHGEF‐6 or Cool‐2) is a member of the Pix family of small Rho GTPase regulators. It functions to ‘turn on’ Rac and Cdc42 activity (Rosenberger G, 2006). αPix has been implicated in hippocampal neuron differentiation (Totaro et al., 2012), while αPix‐null mice have ‘intellectual deficiency’ and reduced Rac activity in the brain (Ramakers et al., 2012). However until now, nothing was known about the role of αPix in the mammary gland or in secretory glandular epithelial cells.

αPix associates with ILK and the IPP complex via Parvin. This occurs through an interaction between the CH1 domain of Parvin and αPix's CH domain (Rosenberger et al., 2003). In vivo work has shown that this interaction is important, as mutations in αPix that prevent its interaction with Parvin cause ‘non‐specific mental retardation’ (Rosenberger et al., 2003; Ramakers et al., 2012).

αPix has also been shown to link ILK with Rac activation. In MDCK epithelial cells, overexpression of the αPix‐binding CH1 domain of Parvin alone increased Rac activity and this was suppressed by a GEF‐deficient version of αPix (Mishima et al., 2004). Similarly, overexpressing ILK in MECs increased Rac activity, which was reduced by overexpression of GEF‐inactive αPix, which suggests a functional role for αPix activity downstream of ILK (Filipenko et al., 2005). Moreover, expression of the K220A or E359 K mutant forms of ILK, which do not bind to Parvin, reduced Rac activity and prevented the co‐IP of an ILK‐Parvin‐Pix complex (Filipenko et al., 2005). The K220A and M mutations also caused perinatal mortality *in vivo*, in knock‐in mice (Lange et al., 2009).

Our results indicating a role for both Parvins and αPix in milk protein expression, suggest that this ILK‐Parvin‐αPix linkage might be significant for tissue‐specific differentiation of epithelial cells. Interestingly, Rac is also essential for mammary epithelial cell differentiation, as we have previously shown that it has a role downstream of integrins and ILK (Akhtar and Streuli, 2006; Akhtar et al., 2009). Future studies will focus on identifying whether αPix regulates Rac activity in 3D cultures of mammary epithelia, and also determining whether αPix is required for lactation in vivo. In addition we will examine whether or not this is the only GEF required for the differentiation response, or if other Rac activators also have a role.

### The adhesive contribution to MEC differentiation and behavior

MECs lacking β1‐Itg or ILK have numerous defects in addition to defective differentiation. Both their ability to form adhesion complexes in 2D culture and polarity in 3D culture is altered (Naylor et al., 2005). However, shαPix‐depleted MECs retain the ability to assemble adhesion complexes and polarize. This suggests that αPix is likely to act downstream of ILK and the IPP complex in MEC differentiation. It has previously been unclear why ILK is required for MEC differentiation, however Rac‐1 acting downstream of ILK influences PrlR signaling via SHP‐2 phosphatase (Akhtar and Streuli, 2006). Future studies will test whether αPix is involved in the activation by Rac of a regulatory kinase that phosphorylates SHP‐2 in the control of PrlR signaling.

ILK‐null MECs display defects in both differentiation and polarity. Interestingly however, Rac1 has a role in tissue‐specific gene expression but not MEC polarity (Akhtar et al., 2009; Akhtar and Streuli, 2013). In this regard, αPix‐depletion was able to phenocopy the Rac1‐null, but not the ILK‐null phenotype in mammary cells. Indeed, our current data show that ILK regulates polarity via the microtubule +TIP‐binding protein EB1.

Thus we hypothesize that there is a bifurcation point for integrin signaling at ILK, whereby αPix and Rac1 (Akhtar and Streuli, 2006) control tissue‐specific gene expression, while a separate pathway downstream of ILK, involving microtubules, controls polarity (Akhtar and Streuli, 2013). These findings support the idea of a signal‐specificity model at adhesion complexes, in which different combinations of scaffold and signaling proteins coordinate to control diverse cellular behaviors.

## Materials and Methods

### Antibodies

Primary antibodies used for immunoblots: Rabbit anti: αParvin (Eurogentec) SK2918, βParvin Proteintech, Manchester, UK Cat no. 14463‐1‐AP, Calnexin Bioquote, York, UK Cat no. SPC‐108A/B, ERK2 Santa Cruz, CA Cat no. SC‐154, GFP Molecular Probes, OR Cat no. A‐11122, ILK Cell signaling, MA Cat no. 3862, phosphoY31 Paxillin Life Technologies, CA Cat no. 44‐720G, phosphoY694 Stat5 Cell Signaling Cat no. 9351, Stat5a Santa Cruz Cat no. SC‐1081 and turboGFP Evrogen, Cambridge, UK Cat no. AB512. Mouse anti: β1‐Integrin BD Transduction, CA Cat no. BD610467, β‐casein (Streuli et al., 1991), Paxillin BD Transduction Cat no. 610052, PINCH BD Transduction Cat no. 612710 and v5 AbD Serotec, Kidlington, UK Cat no. MCA 1360 GA. Secondary antibodies for immunoblots: Anti Mouse (Jackson Immuno Research, Newmarket, UK Cat no. 711‐035‐152) and anti Rabbit (Jackson Immuno Research Cat no. 711‐035‐150) horseradish peroxidase conjugated secondary antibodies. Primary antibodies used for Immunoprecipitation: Mouse anti v5 AbD Serotec Cat no. MCA 1360 GA. Primary antibodies used for Immunofluorescence imaging were the same as for IB except for: ILK clone 65.1.9 a kind gift from Cary Wu. Mouse anti β1‐Integrin (VLA) Merck Millipore, Darmstadt, Germany Cat no. MAB1997, phosphoY397 FAK Life Technologies Cat no. 44‐624, Phospho Histone 3 Merck Millipore Cat no. 06‐570 and Vinculin Sigma–Aldrich, Munich, Germany Cat no. V4505. Secondary antibodies for IF were Alexa Fluor 488 nm or 594 nm conjugated from Life Technologies.

### EpH4 cells

Low passage EpH4 cells were cultured in DMEM F‐12 supplemented with 5% heat inactivated FBS, 5µg/ml Insulin, and 100U/ml Penicillin/streptomycin (EpH4 medium; (Fialka et al., 1996)).

### 3D LrBM culture

Engelbreth‐Holm‐Swarm (EHS) mouse sarcoma basement membrane matrix (Matrigel™, BD Biosciences, Cat no. 354234, Lot no. A7955) was defrosted and spread over dishes, before incubating at 37°C for 1 h. Confluent EpH4 cells were trypsynised and pelleted at 130 rcf for 2 min. The cells were re‐suspended in 1 ml EpH4 medium supplemented with 1 µg/ml hydrocortisone (3D medium) and 3 × 10^5^ cells were plated onto a well of a 12‐well plate coated in LrBM. Medium was changed every 48 h.

### β**‐casein** differentiation assay

EpH4s were grown in 3D LrBM culture for 3–7 days, washed twice in PBS before serum starvation in differentiation medium for 24 h (5 µg/ml Insulin, 1 µg/ml Hydro‐cortisone, 50 U/ml penicillin/streptomycin in DMEM F12 medium). Differentiation was then induced by adding 3 µg/ml sterile filtered sheep pituitary prolactin (Sigma–Aldrich, Munich, Germany, 990 µl ddH_2_O, 10 µl 1M NaOH) for 48–72 h. β‐casein protein and mRNA were detected by immunoblotting and qPCR. The standard work flow for shRNA differentiation assays is outlined in Figure 1C.

### phosphoSTAT5 assay

EpH4s were grown in 3D LrBM culture as described for the β‐casein differentiation assay, before stimulating for 15 min with Prl and lysis in the presence of phosphatase inhibitors (NaF and Sodium Orthovanadate). phosphoY694 STAT5 was detected by immunoblotting.

### Microscopy

For 2D experiments, EpH4s were cultured directly on nitric acid treated glass coverslips. Coverslips were fixed, stained and imaged as described previously (Wang et al., 2011) with a blocking step in 10% goat serum (in PBS) for 1 h at room temperature before primary antibody incubation. For 3D experiments, EpH4s were cultured on LrBM coated coverslips and were fixed for 30 min in 4% PFA and stained and imaged as described previously (Akhtar and Streuli, 2013). Images were processed and analysed using ImageJ (http://rsb.info.nih.gov/ij), and Adobe Photoshop CS4©.

### pH3 proliferation assay

Proliferation was assessed by growing cells on glass coverslips until 80–90% confluent and fixing as described above. Coverslips were stained for phospho‐Histone 3 (pH3). The number of pH3 positive cells were counted and expressed as a % of total cells as indicated by Hoescht stain.

### Lentivirus production and infection

Lentiviruses were produced by transfection of PsPax2 and pMD2G packaging vectors (Tronolab) and the appropriate lentivirus expression vector using PEI (Polyethylenimine) into low passage HEK293T cells cultured in DMEM with Ultra‐glutamine (Lonza, Cat no. BE12/604F/UI), supplemented with 10% FBS (Biosera) and 100 U/ml Penicillin/streptomycin. Lentiviruses were collected and concentrated as described previously (Wang et al., 2011). EpH4s were infected in 2D culture as described previously (Akhtar and Streuli, 2013). Successful infection was determined by live cell visualisation of GFP or mRFP fluorescence.

### FACS

Lentivirally transduced MECs were trypsinised and filtered through a 40 µm filter to generate a single cell suspension. The cells were sorted with a FACS ARIA SORP (BD Biosciences) on the basis of eGFP/tGFP expression (excitation at 488 nm and emission at 530/30) and mRFP expression (excitation at 592 nm and emission at 620/10). FACS data were processed and analysed using DIVA6 software from BD Biosciences. After sorting, the cells were centrifuged at 130 rcf for 2 min and plated at the required density onto plastic or onto LrBM coated dishes.

### Immunoprecipitation

Monoclonal Mouse v5 antibody was bound to protein G magnetic Dynabeads® (Life Technologies) following the manufacturer's instructions. The antibody‐beads complex was cross‐linked by a 30‐min room temperature incubation with rotation in 20 mM Dimethyl Pimelimidate Dihydrochloride dissolved in 200 mM Triethanolamine (both from Sigma–Aldrich). Cross‐linking was quenched by 15‐min room temperature incubation in 100 mM Tris pH 7.5, followed by a low pH shock in 100 mM Glycine pH 3.1 for 2 min. The antibody‐beads complex was blocked by a 30‐min room temperature incubation with rotation in 5% BSA in PBS‐T. Cell lysates were cleared by centrifugation at 14,000*g* for 10 min at 4°C. The cleared lysate was incubated with the antibody‐beads complex for 1 h at room temperature with rotation. Immunoprecipitated complexes were washed three times in 150 mM NaCl wash buffer, then two times in 500 mM NaCl wash buffer (10 mM Tris/Cl pH 7.5, 150–500 mM NaCl, 0.5 mM EDTA, freshly added 1× protease inhibitor cocktail). Proteins were eluted by heating to 65°C in loading buffer and then processed for Western blot analysis.

### Real‐time reverse transcription‐polymerase chain reaction (qPCR)

Total RNA was isolated from adherent cells cultured on Plastic and in LrBM using peqGOLD TriFast™ (peqlab, Cat no. 30–2020). cDNA was synthesized using High Capacity RNA‐to‐DNA Synthesis Kit (Applied Biosystems 4387406). Gene expression was measured using the TaqMan Gene Expression Mastermix (#4369514) and StepOnePlus qPCR machine (Applied Biosystems, Bleiswijk, Netherlands). The recommended TaqMan gene expression assay primer probes (Thermo Fisher, Renfrew, UK) were used for murine αPix (Mm00461751), αParvin (Mm00480444), βParvin (Mm00459990), β‐casein (Mm04207885), and MAPK‐1 (Mm00442479). MAPK‐1 was used as an endogenous control and changes in gene expression were calculated using the ΔΔct method. All qPCR results for β‐casein mRNA are presented in comparison to an assigned control sample treated with Prl, which was normalized to a value of 1. All qPCR results for Pix and Parvin mRNA are presented in comparison to an assigned control sample infected with control lentivirus, which was normalized to a value of 1.

### shRNA

Double stranded oligonucleotides were cloned into pGipz or pLVTHM shRNA expression vectors. The following short nucleotide sequences were used to make shRNAs: pGipz shILK 5′‐AGCTGACATCAATGCAGTGAAT‐3′; pLVshβ1‐itg (Jeanes et al., 2012); pGipz shαPix miR 5′‐AATAGTTGTGTTGTTTACAATA‐3′; pLVshαParvin 5′‐ACGACTGGATCAATGACGTATT‐3′ and pLVshβParvin 5′‐GTCTCTCATCACGTTTGTGAACA‐3′.

### Site directed mutagenesis

Mutagenesis primer pairs were designed to make ILK point mutations; in addition, a ΔN primer was designed to make an ILK construct lacking amino acids 1–63. Mutagenesis was performed following the published QuickChange® II XL Site Directed Mutagenesis kit protocol (Stratagene, CA), using pDual ILKv5eGFP‐F (Boulter et al., 2006) as a template. The primers (Sigma) used were as follows: K220A: 5′‐TGTCGTGGCGGTGCTGAAGG‐3′, K220M: 5′‐TGTCGTGATGGTGCTGAAGG‐3′, E359 K: 5′‐TAGCCCCCAAAGCTCTGCAG‐3′, ΔN: 5′‐GCCGCTAGCAAGCTTATGAACCGTGGGGATGACACC‐3′.

### Statistical analysis

Statistics are from data from three independent experiments. Statistical significance was assessed using a student *t*‐test or ANOVA.
